# Development of breeding lines with three pyramided resistance genes that confer broad-spectrum bacterial blight resistance and their molecular analysis in rice

**DOI:** 10.1186/1939-8433-6-5

**Published:** 2013-02-08

**Authors:** Jung-Pil Suh, Ji-Ung Jeung, Tae-Hwan Noh, Young-Chan Cho, So-Hyun Park, Hyun-Su Park, Mun-Sik Shin, Chung-Kon Kim, Kshirod K Jena

**Affiliations:** Plant Breeding, Genetics, and Biotechnology Division, International Rice Research Institute, DAPO, Box 7777, Metro Manila, Philippines; National Institute of Crop Science, RDA, Suwon, 441-857 Republic of Korea

**Keywords:** Rice, Bacterial leaf blight, Gene pyramiding, Marker-assisted breeding, *Xa4*, *xa5*, *Xa21*

## Abstract

**Background:**

The development of resistant cultivars has been the most effective and economical strategy to control bacterial leaf blight (BB) disease of rice caused by *Xanthomonas oryzae* pv. *oryzae* (*Xoo*). Molecular markers have made it possible to identify and pyramid valuable genes of agronomic importance in resistance rice breeding. In this study, three resistance genes (*Xa4* + *xa5* + *Xa21*) were transferred from an indica donor (IRBB57), using a marker-assisted backcrossing (MAB) breeding strategy, into a BB-susceptible elite japonica rice cultivar, Mangeumbyeo, which is high yielding with good grain quality.

**Results:**

Our analysis led to the development of three elite advanced backcross breeding lines (ABL) with three resistance genes by foreground and phenotypic selection in a japonica genetic background without linkage drag. The background genome recovery of the ABL expressed more than 92.1% using genome-wide SSR marker analysis. The pathogenicity assays of three resistance-gene-derived ABL were conducted under glasshouse conditions with the 18 isolates of *Xoo* prevalent in Korea. The ABL exhibited very small lesion lengths, indicating a hypersensitive reaction to all 18 isolates of *Xoo*, with agronomic and grain quality traits similar to those of the recurrent parent. Pyramiding the resistance genes *Xa4, xa5* and *Xa21* provided a higher resistance to *Xoo* than the introduction of the individual resistance genes. Additionally, the combination of two dominant and one recessive BB resistance gene did not express any negative effect on agronomic traits in the ABL.

**Conclusions:**

The strategy of simultaneous foreground and phenotypic selection to introduce multiple R genes is very useful to reduce the cost and the time required for the isolation of desirable recombinants with target resistance genes in rice. The resistance-gene-derived ABL have practical breeding value without a yield penalty by providing broad-spectrum resistance against most of the existing isolates of BB in South Korea and will have a high impact on the yield stability and sustainability of rice productivity.

**Electronic supplementary material:**

The online version of this article (doi:10.1186/1939-8433-6-5) contains supplementary material, which is available to authorized users.

## Background

Bacterial leaf blight (BB), caused by *Xanthomonas oryzae* pv. *oryzae* (*Xoo*), is a devastating disease in the rice-growing countries of Asia. Infection at maximum tillering stage results in blighting of leaves, which eventually causes significant yield losses in severely infected fields ranging from 20 to 30%, but this can reach as high as 80% (Mew et al.^1992^; Noh et al.^2007^; Shin et al.^1992^). Korean BB isolates have been grouped into five races (K1 to K5) by using five rice cultivars as the *Xoo* differential system (Yun et al.^1985^). Recent pathotyping results indicated that the Korean race K1 has shown a decreasing trend in infection by the spread of rice cultivars with *Xa1* and *Xa3* genes, whereas races K2 and K3 have increased their pathogenicity in Korea (Kim et al.^2009^; Noh et al.^2007^; Shin et al.^1992^). Most of the japonica cultivars possess *Xa1* or *Xa3* or *Xa4* genes for BB resistance, but these genes are showing susceptibility to the new BB strains of Korea (Jeung et al.^2006^; Kim et al.^2009^; Shin et al.^2011^). A new BB race, K3a, that evolved recently caused serious damage to rice production in the southwestern coastal areas of Korea in 2003 (Noh et al.^2003^). Moreover, BB disease is spreading to all regions of Korea because of the effect of climate change and it is causing genetic vulnerability in modern cultivars. Therefore, rice yield has declined and grain quality has decreased by the infection of bacterial blight (Noh et al.^2007^; Shin et al.^1992^).

Breeding and the development of resistant cultivars carrying major resistance (R) genes have been the most effective and economical strategy to control BB disease to have a neutral effect on the environment (Huang et al.^1997^; Jena and Mackill,^2008^; Singh et al.^2001^). Qualitative resistance, which confers major gene-specific resistance against some pathogen races, is the easiest to incorporate into breeding programs and is usually considered a gene-for-gene type of resistance. For many pathogens and insects, this type of qualitative resistance is not often durable because of rapid changes in the virulence in the pathogen or biotype of the population (Leach et al.^2007^). As a result, increasing attention has focused on the accumulation of major disease resistance genes in crop plants. Pyramided lines carrying two, three or four bacterial blight resistance genes showed broad-spectrum and higher resistance than the lines with a single resistance gene (Gu et al.^2005^; Jeung et al.^2006^; Kim et al.^2009^; Singh et al.^2001^; Suh et al.2009a). However, conventional breeding methods to improve rice cultivars for BB resistance have not found much success (Shin et al.^2011^).

To date, at least 38 BB resistance genes conferring host resistance against various strains of *Xoo* have been identified (Bhasin et al.^2012^; Natrajkumar et al.^2012^). All these resistance genes follow a Mendelian pattern of major gene inheritance and express resistance to a diverse group of *Xoo* pathogens (Cheema et al.^2008^; Gu et al.^2005^; Korinsak et al.^2009^; Lee et al.^2003^; Sun et al.^2004^). Several of these genes have already been incorporated into rice cultivars, which are now widely cultivated in many countries (Huang et al.^1997^; Singh et al.^2001^; Sundaram et al.^2008^). Of the 38 R genes, six are physically mapped (*Xa2, Xa4, Xa7, Xa30, Xa33* and *Xa38*) and six are cloned (*Xa1, xa5, xa13, Xa21*, *Xa26* = *Xa3* and *Xa27*) (Bhasin et al.^2012^; Cheema et al.^2008^; Gu et al.^2005^; Liu et al.^2006^; Natrajkumar et al.^2012^; Song et al.^1997^; Sun et al.^2003^; Yang et al.^1998^). BB resistance gene *Xa4* is one of the most widely exploited resistance genes in many rice breeding programs and it confers durable resistance in many commercial rice cultivars (Mew et al.^1992^; Sun et al.^2003^). The *Xa21* gene was identified in the wild species *Oryza longistaminata* and is highly effective against BB races of South and Southeast Asia (Khush et al.^1990^). The *xa5* gene, which is naturally found only within the *Aus* subpopulation of rice (Garris et al.^2003^), provides recessive resistance to several *Xoo* races of the Philippines.

Molecular markers can be used to identify and pyramid favorable (or deleterious) and multiple alleles for biotic and abiotic stress resistance in a collection of diverse genotypes (Jena and Mackill,^2008^; Lee et al.^2003^; Singh et al.^2001^; Suh et al.2009a). Marker-assisted selection (MAS) for pyramiding important genes along with rapid background recovery of the recurrent parent, while maintaining the exquisite quality characteristics of rice, could be an effective approach for rice improvement (Shanti et al.^2010^; Singh et al.^2001^; Suh et al.2009a; Suh et al.^2011^; Sundaram et al.^2008^; Xu and Crouch,^2008^; Ye 2010). Gene pyramiding is difficult using conventional breeding methods due to the dominance and epistasis effects of genes governing disease resistance. Moreover, genes with similar reactions to two or more races are difficult to identify and transfer through conventional approaches (Joseph et al.^2004^; Rajpurohit et al.^2011^; Sundaram et al.^2009^). However, the availability of molecular markers closely linked to each of the resistance genes makes the identification of plants with two and three genes possible (Shanti et al.^2010^; Singh et al.^2001^; Sundaram et al.^2008^). Three BB resistance genes (*xa5, xa13* and *Xa21*) were pyramided in cultivar PR106 using MAS. Testing with 17 *Xanthomonas oryzae* pv. *oryzae* (*Xoo*) isolates under artificial inoculation and field conditions showed that the combination of genes provided a wider spectrum of resistance to the pathogen populations prevalent in the region (Singh et al.^2001^). In a previous study, the IR24 NILs (IRBB lines) containing *Xa4*, *xa5*, *Xa7* and *Xa21* genes and their combinations conferred different degrees of resistance to K1, K2, K3 and K3a races in a field inoculation experiment in Korea (Jeung et al.^2006^; Kim et al.^2009^; Suh et al.2009a). The resistance gene pyramid of *Xa4* + *xa5* + *Xa21* would be the most effective strategy for improving Korean japonica cultivars for BB resistance (Jeung et al.^2006^; Kim et al.^2009^). The identification of closely linked markers has also enabled pyramiding of *Xa4*, *xa5* and *Xa21* using MAB.

This study reports a successful transfer of bacterial leaf blight resistance genes *Xa4, xa5* and *Xa21* from indica rice into an elite japonica rice cultivar using MAB and marker-assisted background analysis of selected BC progenies using SSR markers.

## Results

### Transferring BB resistance genes by MAB

**Figure 1:**
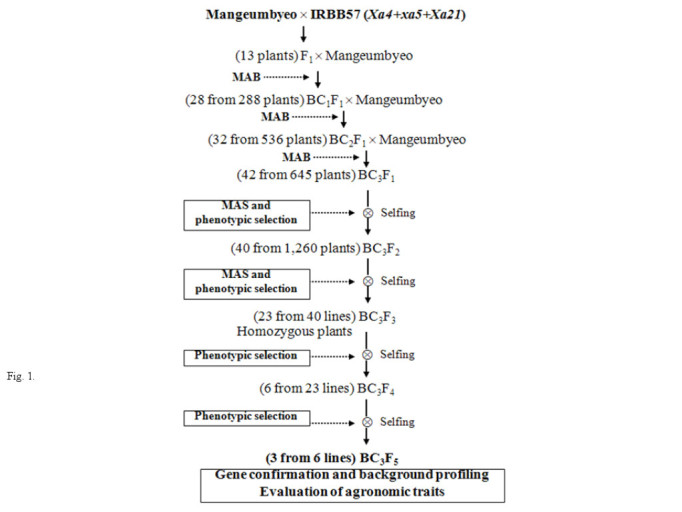
**Scheme for the development of** ***Xa4, xa5*** **and** ***Xa21*** **gene-pyramided backcross breeding lines using marker-assisted foreground and background selection.**

**Figure 2:**
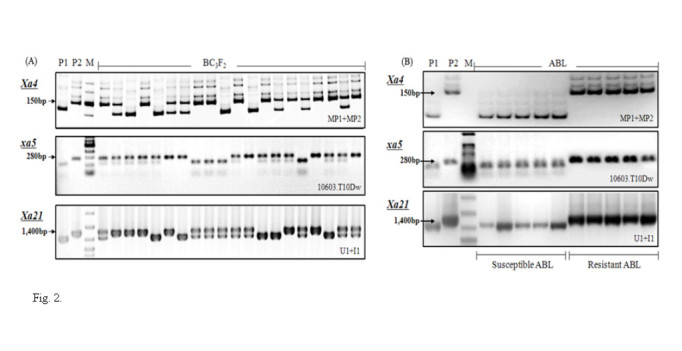
**PCR analysis of the parental lines and BC**_**3**_**F**_**2**_**plants (A) and resistance gene confirmation of advanced backcross breeding lines (B).** DNA amplified with primers MP1 + MP2, 10603.T10Dw (digested with *Rsa* I) and U1/I1 was linked with resistance genes *Xa4, xa5* and *Xa21*, respectively. P1: Mangeumbyeo, P2: IRBB57, M: DNA ladder marker.

F_1_ plants with heterozygous alleles of the three BB resistance genes (*Xa4, xa5* and *Xa21*) were obtained from the cross of Mangeumbyeo and IRBB57. They were confirmed for their heterozygosity by DNA analysis of markers linked with the three R genes and were backcrossed with Mangeumbyeo as the female parent. A total of 288 BC_1_F_1_ progenies were produced and individual plants heterozygous at the *Xa4, xa5* and *Xa21* loci were identified and used for further backcrossing with the recurrent parent. Of the 288 BC_1_F_1_ plants that were analyzed with three STS markers, 28 plants were selected as having an allele of three resistance genes on the basis of molecular marker analysis and phenotypic selection. The advanced backcross progenies of BC_2_ and BC_3_ were obtained from the crosses of selected resistant BC_1_F_1_ (28 plants from 288 plants), BC_2_F_1_ (32 plants from 536 plants) and BC_3_F_1_ (42 plants from 645 plants) plants based on the dual-selection procedure of the BB-resistant phenotype and foreground selection using the *Xa4, xa5* and *Xa21* gene-specific DNA markers (Figure 1). Progenies of the BC_3_F_1_ generation were advanced by dual-selection and selfing, and promising BB-resistant breeding lines were developed. Phenotypic selection at each backcross and selfing generation was conducted to eliminate plants with linkage drag traits such as high sterility, tall plant type and late flowering. Thus, the population size for MAS could be reduced as we removed the plants with an undesirable phenotype. We selected three ABL from BC_3_F_5_ progenies based on their reaction to selected BB isolates and the presence of homozygous marker alleles for the BB resistance genes and desirable agronomic traits (Table 1 and Figure 2).

**Table 1:** **List of three advanced backcross breeding lines, check varieties and recurrent and donor parents used in this study**

Cultivar/breeding line	Description (generaton)^z^	Cross	Gene	Remarks
Mangeumbyeo	Recurrent parent	Milyang71/Saikai PL1	Unknown	Korean elite japonica variety
IRBB57	Donor parent	IR24/*Xa4 + xa5 + Xa21*	*Xa4 + xa5 + Xa21*	Multi-R-gene NILs (indica)
SR30075-1-1-13-3-1-26-1	ABL4225 (BC_3_F_5_)	Mangeumbyeo*4/IRBB57	*Xa4 + xa5 + Xa21*	Mangeumbyeo genetic background
SR30075-1-1-13-40-1-26-1	ABL4228 (BC_3_F_5_)	Mangeumbyeo*4/IRBB57	*Xa4 + xa5 + Xa21*	Mangeumbyeo genetic background
SR30075-2-1-3-25-1-1-1	ABL4242 (BC_3_F_5_)	Mangeumbyeo*4/IRBB57	*Xa4 + xa5 + Xa21*	Mangeumbyeo genetic background
IRBB4	Check	IR24/TKM6	*Xa4*	Near-isogenic line for BB (indica)
IRBB5	Check	IR24/DZ192	*xa5*	Near-isogenic line for BB (indica)
IRBB21	Check	IR24/*O. longistaminata*	*Xa21*	Near-isogenic line for BB (indica)

^z^ ABL: advanced backcross breeding lines having *Xa4 + xa5 + Xa21* genes.

### Evaluation of BB resistance

The ABL with three resistance genes were evaluated for their resistance to BB under glasshouse conditions with the 18 isolates of *Xoo* prevalent in Korea. One of these isolates, HB01009 belongs to race K3a, a widely distributed *Xoo* pathotype in the southwestern coastal areas of Korea (Noh et al.^2003^). The lesion lengths obtained after inoculation with these isolates are shown in Table 2. Mangeumbyeo was highly susceptible to all isolates, with lesion length ranging from 9 to 18.2 cm, whereas donor line IRBB57 pyramided with the R genes *Xa4, xa5* and *Xa21* was highly resistant against all isolates, with lesion length of <0.5 cm. Compared to Mangeumbyeo, the leaves of the NILs with *Xa4, xa5* and *Xa21* genes showed susceptible, moderately resistant and resistant reactions to the BB strains. However, the ABL with *xa4 + xa5 + xa21* pyramided genes in the Mangeumbyeo background exhibited very small lesion lengths, indicating very high resistance to all 18 isolates of *Xoo*, with average lesion lengths being <0.3 cm. Our results indicated that the genes in combinations were more effective against the pathogen than a single gene (Table 2). Resistance genes *xa5* and *Xa21* were effective against 14 of the isolates from Korea used in this study, whereas resistance gene *Xa4* was resistant to 8 isolates only. Based on this result, we infer that, individually, *xa5* and *Xa21* were more effective resistance genes than *Xa4*.

**Table 2:** **Average lesion length in centimeters of advanced backcross breeding lines, near-isogenic lines carrying single bacterial blight resistance genes and recurrent and donor parents against each of 18 Korean** ***Xanthomonas oryzae*** **pv.** ***oryzae*** **isolates**

Isolate	RP^y^	DP^y^	IRBB4	IRBB5	IRBB21	ABL4225^z^	ABL4228	ABL4242
HB01009	13.0	0.1	2.8	0.5	1.0	0.3	0.6	0.4
HB02010	14.1	0.1	2.5	0.1	15.0	0.1	0.1	0.1
HB02024	13.0	0.3	2.0	2.5	1.0	0.1	0.2	0.1
HB02038	10.2	0.1	9.0	7.0	1.5	0.1	0.1	0.4
HB03034	9.0	0.2	8.0	1.5	7.5	0.5	0.1	0.3
HB03055	9.5	0.2	2.1	1.5	7.5	0.7	0.2	0.1
HB04024	18.2	0.1	2.3	7.5	1.5	0.1	0.1	0.1
HB04030	14.0	0.1	7.5	1.5	1.2	0.1	0.1	0.1
HB04032	10.3	0.1	8.5	2.5	1.5	0.1	0.1	0.2
HB04040	14.0	0.5	9.0	2.5	2.0	0.3	0.4	0.1
HB04052	13.0	0.1	9.5	2.5	1.8	0.1	0.7	0.1
HB04064	15.4	0.1	10.5	1.5	16.0	0.1	0.1	0.1
HB04079	15.4	0.1	11.5	1.5	3.0	0.5	0.1	0.1
HB04084	12.2	0.1	3.5	7.5	3.0	0.1	0.5	0.5
HB04087	10.0	0.3	2.5	7.0	1.0	0.4	0.5	0.5
HB05004	18.0	0.1	9.5	2.0	1.5	0.7	0.8	0.1
HB05027	13.5	0.1	7.5	2.0	1.0	0.7	0.1	0.1
HB05029	17.2	0.1	2.5	1.5	2.0	0.1	0.2	0.1

^y^ RP (Recurrent parent): Mangeumbyeo, DP (Donor parent): IRBB57.

^z^ABL: advanced backcross breeding lines having *Xa4 + xa5 + Xa21* genes.

### SSR-based genetic background profiling of ABL

**Figure 3:**
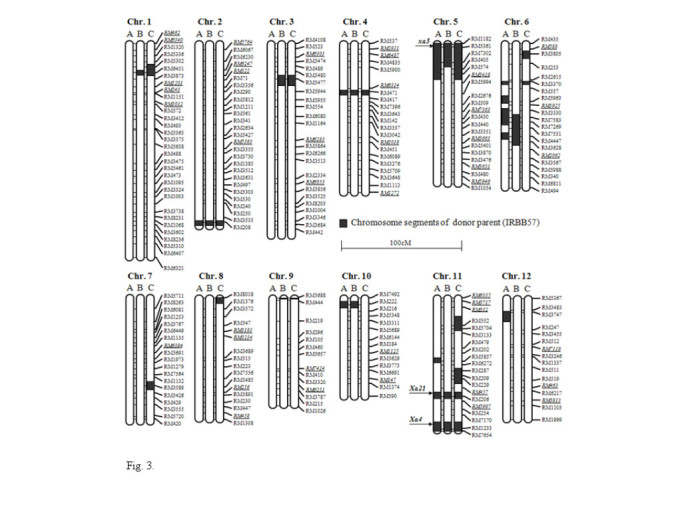
**Background selection of three advanced backcross breeding lines (ABL) in Mangeumbyeo genetic background.** Letters **A**, **B** and **C** on top are ABL4225, ABL4228 and ABL4242, respectively. The black box indicates substituted chromosome segments of the donor parent in ABL.

A total of 248 SSR markers were used for background selection of the three ABL along with the BB-resistant donor line IRBB57 and a genetic map covering a 1,446.6 cM region of the *O. sativa* genome was constructed (Figure 3). The marker polymorphisms between Mangeumbyeo and IRBB57 were 83%. Each ABL contains an SSR marker-defined chromosome segment from the donor in the genetic background of the recurrent parent, Mangeumbyeo. The average percentage of donor parent chromosome substitution in ABL4225, ABL4228 and ABL4242 was 7, 5.5 and 7.9%, respectively (Table 3). The substituted chromosome segments in ABL were distributed around the regions of *xa5* located on chromosome 5 and *Xa4* and *Xa21* located on chromosome 11. In our study, ABL4228 inherited the smallest size (5.5%) of the substituted chromosome segments from the donor genotypes.

**Table 3:** **Simple sequence repeat markers with polymorphism between the recurrent parent and the donor parent and substituted chromosome segments from donor parent in advanced backcross breeding lines of rice**

Chr. no.^v^	No. of markers	Chr. length (cM)^w^	Interval (cM)^x^	PM (%) of RP/DP^y^	Chromosome segments of DP (%)^z^		
					ABL4225	ABL4228	ABL4242
1	32	181.5	5.7	84.4	2.0	2.8	10.5
2	27	151.6	5.6	85.2	1.2	1.2	1.2
3	25	157.9	6.3	88.0	0.0	5.4	5.4
4	21	126.5	6.0	76.2	3.2	3.2	3.2
5	21	118.0	5.6	76.2	25.2	16.1	25.2
6	21	122.7	5.8	85.7	25.5	22.0	6.8
7	19	94.1	5.0	94.7	0.0	0.0	6.8
8	17	103.2	6.1	76.5	0.0	0.0	4.7
9	14	91.3	6.5	85.7	0.0	1.3	1.3
10	15	83.8	5.6	86.7	7.0	7.0	0.0
11	20	112.9	5.6	75.0	11.3	6.8	29.8
12	16	103.1	6.4	81.3	8.2	0.0	0.0
Average (total)	(248)	(1446.6)	5.9	83.0	7.0	5.5	7.9

^v^ Chromosome number.

^w^ Chromosome length in centiMorgan (cM).

^x^ Average marker interval.

^y^ Polymorphism between Mangeumbyeo (RP; recurrent parent) and IRBB57 (DP; donor parent).

^z^ ABL4225, ABL4228 and ABL4242: advanced backcross breeding lines in Mangeumbyeo genetic background.

### Agronomic traits and grain quality performance of ABL

The agronomic traits of ABL evaluated in the field and laboratory showed that most of the morphological traits, including plant type and grain quality, were similar to those of the recurrent parent, Mangeumbyeo (Table 4). Traits such as days to heading, panicle number, grain yield, 1,000-grain weight of brown rice, amylose content and alkali digestion value of milled rice, protein content of brown rice and alkali digestion value of the selected three ABL were almost the same as those of Mangeumbyeo. However, the DTH of ABL4225 and ABL4228 were 12–13 days less than those of Mangeumbyeo. The culm length of the three ABL was shorter by 4–8 cm than that of Mangeumbyeo. This is a desirable agronomic trait for lodging resistance, thus reducing yield loss. The grain yield of ABL did not show a significant difference from Mangeumbyeo even though the number of grains per panicle of the three ABL was more than that of the recurrent parent. This may be due to a reduction in spikelet fertility per se and number of grains per panicle. All of the ABL were recovered with japonica grain characteristics of the recurrent parent with a non-chalky appearance and similar values for AC, PC, ADV and grain shape (short grain type), having homozygous alleles of the *Xa4, xa5* and *Xa21* genes (Table 4).

**Table 4:** **Performance of principal agronomic and grain quality traits of three ABL, which were selected as the most promising lines**

Variety	DTH^z^	CL (cm)	PL (cm)	PN	NGP	FER (%)	GY (t/ha)	GW (g)	L/W	AC (%)	PC (%)	ADV (1–7)
Mangeumbyeo	115b	81 cd	19a	15a	108b	96b	7.86ab	19.9a	1.85	20.8a	6.3a	6.8b
IRBB57	115b	67a	23b	14a	128c	92a	7.57a	22.5b	3.04	24.5b	7.3b	2.0a
ABL4225	102a	73b	21ab	14a	118b	94ab	7.70ab	18.7a	1.74	19.9a	6.8ab	6.7b
ABL4228	103a	75bc	19a	15a	117b	93a	7.81ab	18.2a	1.78	19.1a	6.7ab	6.6b
ABL4242	114b	77bcd	20a	15a	120bc	95ab	7.98b	19.1a	1.74	20.5a	6.5ab	6.8b

^z^ DTH: days to heading, CL: culm length (cm), PL: panicle length (cm), PN: panicle number, NGP: number of grains per panicle, FER: fertility of spikelets (%), GY: grain yield (t/ha), GW: 1,000-grain weight of brown rice (g), L/W: ratio of seed length/width, AC: amylose content of milled rice (%), PC: protein content of brown rice (%), ADV: alkali digestion value (1–7), and a higher value indicates better quality. Means followed by the same letter are not significant at the 5% significance level by the least significant difference test (LSD = 0.05).

## Discussion

Most japonica rice cultivars exhibit high susceptibility to BB disease, except to race K1 in Korea, because of their narrow genetic diversity. It is imperative to develop new BB-resistant rice cultivars with high yield potential and grain quality using modern tools of biotechnology. However, it is often difficult to introduce the BB resistance genes from indica germplasm sources into a japonica genetic background by conventional breeding methods due to the unexpected linkage drag. Pyramiding resistance genes is difficult to accomplish using conventional breeding because of the dominance and epistasis effects of the genes controlling disease resistance. Nevertheless, using the tools of biotechnology, it is possible to transfer or pyramid valuable genes of BB resistance into rice without linkage drag (Rajpurohit et al.^2011^; Shanti et al.^2010^; Singh et al.^2001^; Sundaram et al.^2008^). Mangeumbyeo is a japonica cultivar with good grain and cooking quality and high yield potential but it is highly susceptible to BB races. An IRBB57 NIL carrying *Xa4*, *xa5* and *Xa21* genes in an IR24 genetic background conferred strong resistance to all Korean BB races, including K3a (Jeung et al.^2006^; Suh et al.2009a). We have introduced the three BB resistance genes (*Xa4* + *xa5* + *Xa21*) from IRBB57 into Mangeumbyeo through simultaneous foreground and phenotypic selection. Eventually, it was possible to introduce three BB resistance genes with desirable agronomic traits using marker-assisted backcrossing. All three co-dominant molecular markers linked to the target genes (*Xa4*, *xa5* and *Xa21*) were used for MAB and the markers were polymorphic between the donor parent IRBB57 and recurrent parent Mangeumbyeo. The validated markers could thus be used successfully to pyramid and confirm the three resistance genes in advanced backcross lines. Finally, we also analyzed the genetic background of the three selected ABL (BC_3_ progenies) with high background genome recovery. Conventional backcross breeding has difficulty in confirming the several resistance genes combined in breeding lines using phenotypic selection with *Xoo* inoculation (Rajpurohit et al.^2011^; Shanti et al.^2010^; Sundaram et al.^2008^). The best strategy to pyramid or introduce multiple genes and recover a maximum recurrent parent background effect in the shortest time will be to take up the transfer of genes simultaneously, generate a large backcross population and select the target genes through foreground selection and flanking marker analysis to reduce the persistent linkage drag (Rajpurohit et al.^2011^; Ye,^2010^). However, if we select backcross lines with target genes using molecular markers, linkage drag often occurs in indica/japonica. So, we selected the backcross progenies in each backcross and segregating generation through foreground and phenotypic selection simultaneously to reduce the linkage drag. This expensive, cumbersome and time-consuming background selection can be avoided and substituted by another backcross with the recurrent parent, if necessary. Final backcross progenies could be confirmed with the substituted chromosome segments by background analysis using genome-wide molecular markers. On the basis of comprehensive foreground selection, phenotypic selection for morphological and quality traits, and background genotyping, three BC_3_F_5_ gene-pyramid lines with pyramided genes homozygous at all three target loci were derived from the donor parent. The three R-gene-derived ABL exhibited high resistance upon inoculation with *Xoo* strains and had nearly the average expected 93.75% background genome recovery.

In an earlier study, it was reported that the favorable characteristics of Pusa Basmati 1 with two BB resistance genes could be recovered using MAS just in BC_1_ because of stringent phenotypic selection without any background selection only in segregating generations (Joseph et al.^2004^). Similarly, BC_4_ pyramided lines of Sambha Mahsuri with three BB resistance genes (*xa5, xa13* and *Xa21*) were developed by simultaneous foreground and background selection and the selected lines recovered 97% recurrent parent background, exhibiting a broad-spectrum resistance against multiple *Xoo* isolates (Sundaram et al.^2008^). In this study, we selected elite ABL with three BB resistance genes in the BC_3_ generation because BC_1_ and BC_2_ progenies were having some undesirable phenotypic traits such as awns, shattering and spikelet sterility. It is possible to recover the recurrent parent phenotype in one or two backcrosses if we introduce multiple resistance genes from indica to indica cultivars (Joseph et al.^2004^; Rajpurohit et al.^2011^; Singh et al.^2001^) and we may also need at least two backcrosses to introduce one resistance gene from indica to japonica cultivars (Suh et al.2009b; Suh et al.^2011^). However, our results suggest that at least three backcrosses are essential to recover the phenotype of the recurrent parent if multiple resistance genes such as *Xa4, xa5* and *Xa21* are transferred from an indica cultivar into a japonica cultivar for broad-spectrum BB resistance.

Three BB resistance-gene-derived ABL were evaluated for their resistance to BB under glasshouse conditions with the 18 isolates of *Xoo* prevalent in Korea. One of these isolates, called HB01009, belongs to the new race K3a (Noh et al.^2003^). The *Xa21* and *xa5* genes and their combinations conferred strong resistance to the K3a isolate (Suh et al.2009a,2009b). Variable reactions of the *Xoo* isolates to *Xa4*, *xa5* and *Xa21* suggest that *xa5* and *Xa21* are more effective in resistance to 14 isolates than *Xa4* because *Xa4* showed resistance to 8 isolates only. However, the cumulative effect of the three resistance genes (*Xa4 + xa5 + Xa21*) in the ABL in the Mangeumbyeo genetic background exhibited very high resistance to all 18 isolates of *Xoo,* including the most virulent isolate of race K3a. The results indicated that the genes in combinations were more effective against the pathogen strains than a single resistance gene alone. The resistance appears to be more durable if different resistance genes are combined (Jeung et al.^2006^; Kim et al.^2009^; Singh et al.^2001^; Suh et al.2009a). This indicates that there is some kind of quantitative complementation with the presence of multiple resistance genes having an additive effect on the overall level of resistance. Accumulating major genes for resistance in an elite genotype by conventional breeding is laborious, time-consuming and very difficult when two or more of the resistance genes are pyramided into an elite cultivar. However, marker-assisted backcrossing with accurate phenotypic selection is the most effective method for a selective transfer or pyramiding of resistance genes into elite rice cultivars free from linkage drag, eventually restoring the recurrent parent genotype (Joseph et al.^2004^; Shanti et al.^2010^; Singh et al.^2001^; Suh et al.^2011^). The ABL with the three resistance genes in combination have a practical breeding value by providing a wider spectrum of resistance against most of the existing BB isolates in the region and will have a high impact on the yield stability and sustainability of the rice crop in the region. The grain quality characteristics of the three resistance-gene-derived ABL are not significantly different from those of the parent Mangeumbyeo. This indicates that the BB resistance-gene combinations are not closely linked with any negative allele controlling grain quality. It is also reported that *Xa1*, *Xa2* and *Xa3* genes have no negative effect for the traits associated with grain quality and the taste of cooked rice (Shin et al.^2006^). The recurrent parent greatly influenced the determination of grain quality, milling characteristics and cooking and eating qualities. Therefore, the choice of the recurrent parent plays a critical role in backcross breeding programs (Shin et al.^2006^; Ye^2010^). The yield and agronomic traits of the ABL in this study are also similar to those of Mangeumbyeo, indicating that there is no apparent agronomic trait penalty associated with the presence of the resistance genes.

In our study, an additional backcross with the recurrent parent was required to recover the desirable phenotype in the BC_3_ progenies. Three BC_3_F_5_ progenies were mostly homozygous for the target traits based on MAS with agronomic traits similar to those of the recurrent parent, Mangeumbyeo, with high resistance to bacterial blight. The background genotype recovery varied from 92.1 to 94.5%. Even though the three ABL showed highly recovered chromosome segments, they could not exhibit a similar phenotype with the recurrent parent because the insertion of small chromosome segments also affected phenotype. Theoretically, with three backcrosses, the average background genotype recovery should be 93.75%, a background recovery rate similar to that of the selected ABL in this study. On the contrary, the background recovery of the recurrent wheat parent during the introgression of stripe rust resistance without marker-assisted background selection was only 82% in BC_4_F_7_ progenies (Randhawa et al.^2009^). However, 97% of the background genotype was obtained in BC_2_F_2:3_ progenies by using foreground selection of the target traits, background selection for flanking markers, non-carrier chromosome markers and whole-marker screens during two successive backcrosses in a large backcross population. A high rate of background genotype recovery of the recurrent parent was 86.72% in the BC_1_F_3_ generation using MAS and phenotypic selection during the introgression of two BB resistance genes in indica/indica crosses (Joseph et al.^2004^). In our study, a similar strategy of simultaneous foreground and phenotypic selection was followed for higher background genotype recovery in the japonica/indica cross in three backcrosses. This approach is very useful to reduce the cost and time required for the recovery of desirable recombinants to a considerable extent with target resistance genes in japonica/indica crosses. Therefore, it can be directly developed in a commercial variety. Introgression of resistance with a penalty in yield and grain quality characters would be a futile exercise, as the developed lines would not be accepted by farmers. The three-gene pyramided ABL developed in our study without a penalty in yield and grain quality would be of great advantage to rice farmers in BB-endemic rice areas.

## Conclusions

Host-plant resistance is a cost-effective and environmentally safe approach to reduce yield loss caused by BB disease of rice. Several BB resistance genes identified to date are either race specific or express susceptibility to the emerging races of the pathogen. Our study provides some clues to a successful pyramiding of three BB resistance genes into an elite japonica cultivar to control BB disease caused by a new race, K3a. We used a dual- selection strategy of phenotypic and genotypic selection along with background genotyping to isolate improved breeding lines with three pyramided genes conferring strong resistance to BB. Furthermore, our study on the evaluation of agronomic traits revealed that the accumulation of three-gene pyramids did not show a yield penalty. Future studies on the transfer of these pyramided genes into other genetic backgrounds may help in controlling BB disease caused by different races of the pathogen.

## Methods

### Plant materials used

IRBB57, a near-isogenic line in the background of IR24 possessing a combination of three genes (*Xa4* + *xa5* + *Xa21*), was used as the donor parent for transferring BB resistance genes into japonica rice cultivars. Mangeumbyeo, a BB-susceptible elite japonica cultivar with good grain quality, was used as the recurrent parent. A cross was made between Mangeumbyeo and IRBB57, which carries three BB resistance genes. F_1_ plants were backcrossed with the recurrent parent. Advanced backcross breeding lines (ABL) in a japonica genetic background were developed by the marker-assisted backcross (MAB) breeding strategy. Among the BC_1_F_1_ plants, polymerase chain reaction (PCR)-based molecular markers linked to *Xa4, xa5* and *Xa21* were used to select plants with resistance alleles. A similar strategy was used in the BC_2-3_ F_1_ to obtain BC_3_F_2_ populations from which the introduced R genes were selected. The BC_3_F_2_ plants were selfed and advanced generation progenies were produced on the basis of marker-assisted selection (MAS) and were inoculated with BB isolates/races, including the K3a isolate. The selected and confirmed ABL were used for overall resistance evaluation and background profiling (Table 1).

### Bacterial blight inoculation and evaluation

The parents and segregating ABL materials were grown in the glasshouse of the National Institute of Crop Science (NICS). At the maximum tillering stage, the plants were inoculated with the K3a isolate (HB01009) of *Xanthomonas oryzae* pv. *oryzae* (*Xoo*) using the leaf clipping method (Kauffman et al.^1973^). Plant reaction to the disease was scored 14 days after inoculation by measuring lesion length (cm). The reaction of resistance was expressed in lesion length (resistant: < 3 cm, moderately resistant: 3–5 cm, susceptible: > 5 cm) (Jeung et al.^2006^). The selected ABL confirming three resistance genes were inoculated with 18 predominant *Xoo* isolates from Korea (Table 2).

### Resistance gene confirmation by DNA markers

Genomic DNA was extracted from fresh frozen leaves of rice plants using the CTAB method with little modification (Murray and Thompson^1980^). Three gene-specific PCR markers, MP1 + MP2, 10603.T10Dw and U1/I1, tightly linked to the resistance genes *Xa4, xa5* and *Xa21*, respectively, were used to confirm the presence of the R genes in each backcross generation (Table 5). PCR was performed in a total volume of 20 μl containing 40 ng of DNA template, 10 pmole of each primer, 1.5 mM of MgCl_2_, 0.2 mM of dNTP and 1U of *Taq* polymerase (Suh et al.2009a). The PCR amplification condition was with one cycle at 95°C for 4 min, followed by 35 cycles at 95°C for 30 s, at 56°C (MP1 + MP2 and U1/I1) or 65°C (10603.T10Dw) for 30 s and at 72°C for 1 min, with a final extension at 72°C for 10 min (Bio-Rad, PTC-200 Thermocycler; Germany). Marker allele types of the genotypes were determined based on the unique band sizes as well as the banding patterns derived from PCR products (MP1 + MP2 and U1/I1) or from cleaved PCR products (10603.T10Dw) by *Rsa* I enzyme, for which 4 μl of the PCR product was digested by 2.5 U of restriction endonuclease in a 20 μl reaction volume at 37°C for 3 hours. Agarose gel (1.5%, 0.5×TBE, 150 V) and natural polyacrylamide gel (8% polyacrylamide, 0.5×TBE, 200 V) electrophoresis were used for the PCR products from 10603.T10Dw (treated by *Rsa* I) and U1/I1 primers, and the PCR products from MP1 + MP2, respectively, and stained by ethidium bromide to visualize the DNA.

**Table 5:** **Gene-specific polymerase chain reaction primers used for the identification of major BB resistance genes**

Resistance gene	Chr.no.	Marker name	Primer sequences used for gene detection		Expected size (bp)	Band type	Reference
			Forward (5′-3′)	Reverse (5′-3′)			
*xa5*	5	10603.T10Dw	GCACTGCAACCATCAATGAATC	CCTAGGAGAAACTAGCCGTCCA	280	Co-dominant	Jeung et al. (Unpublished)
*Xa4*	11	MP1 + MP2	ATCGATCGATCTTCACGAGG	TGCTATAAAAGGCATTCGG	150	Co-dominant	Sun et al.^2003^
*Xa21*	11	U1/I1	CGATCGGTATAACAGCAAAAC	ATAGCAACTGATTGCTTGG	1,400	Co-dominant	Wang et al. 1996

### Background profiling by SSR marker analysis

A total of 248 SSR markers of known chromosomal positions distributed evenly on the 12 chromosomes with an average marker interval of 5.9 cM were used in a genome-wide survey to identify the chromosome segment substitution locations in the three ABL compared with the donor line. The SSR markers polymorphic between the two parents were used for background genotyping to recover the recipient parent genome. The lengths of substituted chromosome segments in ABL were estimated based on the graphical genotyping procedure (Suh et al.2009b; Xi et al.^2006^). A chromosome segment flanked by homozygous marker alleles of the donor parent was considered a 100% donor type, a chromosome segment flanked by homozygous marker alleles of the recipient parent was considered a 0% donor type and a chromosome segment flanked by one marker allele of the donor parent and another marker allele of the recipient parent was considered a 50% donor type. The linkage and orientation of SSR markers on chromosomes were assigned following the SSR map constructed by McCouch et al. (2002) and as depicted in Gramene (http://www.gramene.org/).

### Agronomic and grain quality evaluation of the ABL

The parents and the three ABL were planted in a four-row plot with 35 plants per row by 30×15-cm spacing in a randomized complete block design with three replications and were evaluated for agronomic traits in the rice experimental plot of NICS, Suwon, Korea, using the standard evaluation method of rice (RDA Rural Development Administration^2003^). The amount of standard fertilizer application in the experimental field was N-P_2_O_5_-K_2_O = 90-45-57 kg/ha. Commercial pesticides were applied for the protection of plant materials. For each line, five plants in the middle rows were used to determine days to heading (DTH), culm length (CL), panicle number (PN), panicle length (PL), number of grains per panicle (NGP), fertility of spikelets (FER), 1,000-grain weight of the brown rice (GW), ratio of seed length/width (L/W) and grain yield (GY; t/ha). DTH was evaluated as the number of days from sowing in the field until 50% heading of the panicles in the plants. CL was calculated as the average number in centimeters from the ground to the neck of the tallest panicle. PL was measured as the average number in centimeters from the panicle neck to the panicle tip based on an evaluation of all the panicles from the plants. PN was the average number of panicles on the plants. NGP was calculated by counting the total number of filled spikelets from the plants. FER was calculated as a percentage: the number of filled spikelets divided by the number of spikelets per panicle. GW was measured in grams as the average weight of 1,000 fully filled brown rice grain from each plant.

Grain yield per plot was evaluated based on a grain harvest of 100 plants in the central row of each plot. Grain quality was estimated for alkali digestion value (ADV), amylose content of milled rice (AC), protein content of brown rice (PC) and chalkiness of brown rice (CK; 0: non-chalkiness, 3: high chalkiness). ADV was evaluated based on the procedure of Little et al. (1998). AC was determined by the relative absorbency of starch-iodine color in a digested solution of 100-mesh rice flour by Juliano’s (Juliano^1973^) modified method. PC was calculated by total nitrogen multiplied by 5.95 after determining the nitrogen content of rice material using the Micro-Kjeldahl method (Foss: 2300 Kjeltec Analyzer). The least significant difference (LSD) and Duncan’s multiple range test (DMRT) were used for multiple mean comparisons using the SAS statistical analysis software (version 8.2; SAS Institute, Cary, NC).

## Authors’ original submitted files for images

Below are the links to the authors’ original submitted files for images. Authors’ original file for figure 1 Authors’ original file for figure 2 Authors’ original file for figure 3
