# Supplementary figures and images for: Development of breeding lines with three pyramided resistance genes that confer broad-spectrum bacterial blight resistance and their molecular analysis in rice
Source: Rice (N Y). 2013 Feb 8;6:5. doi: 10.1186/1939-8433-6-5 (PMC4883717; doi:10.1186/1939-8433-6-5)

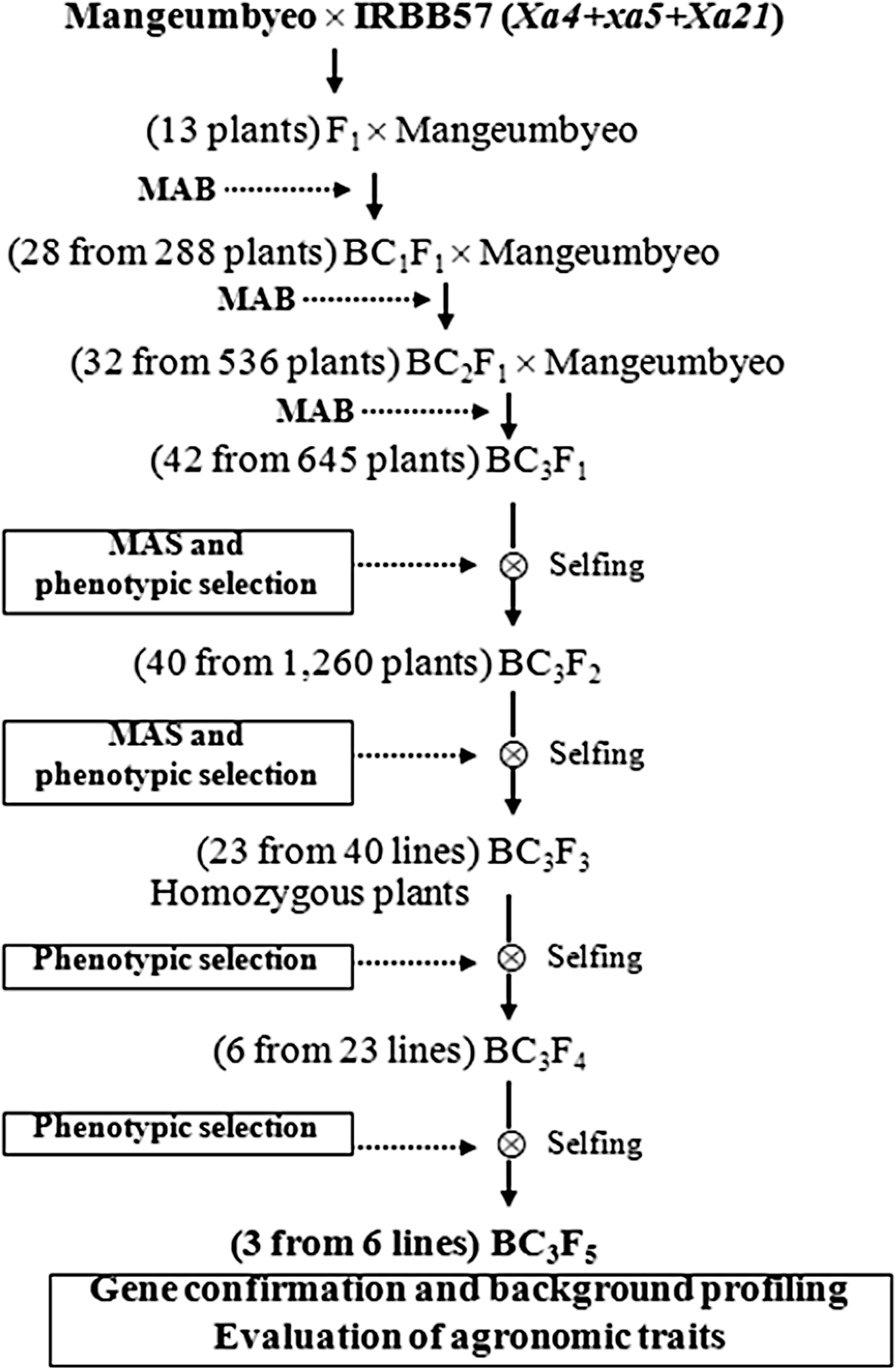

Supplement: Supplementary file 1 — Authors’ original file for figure 1 [file 12284_2012_42_MOESM1_ESM.tiff]

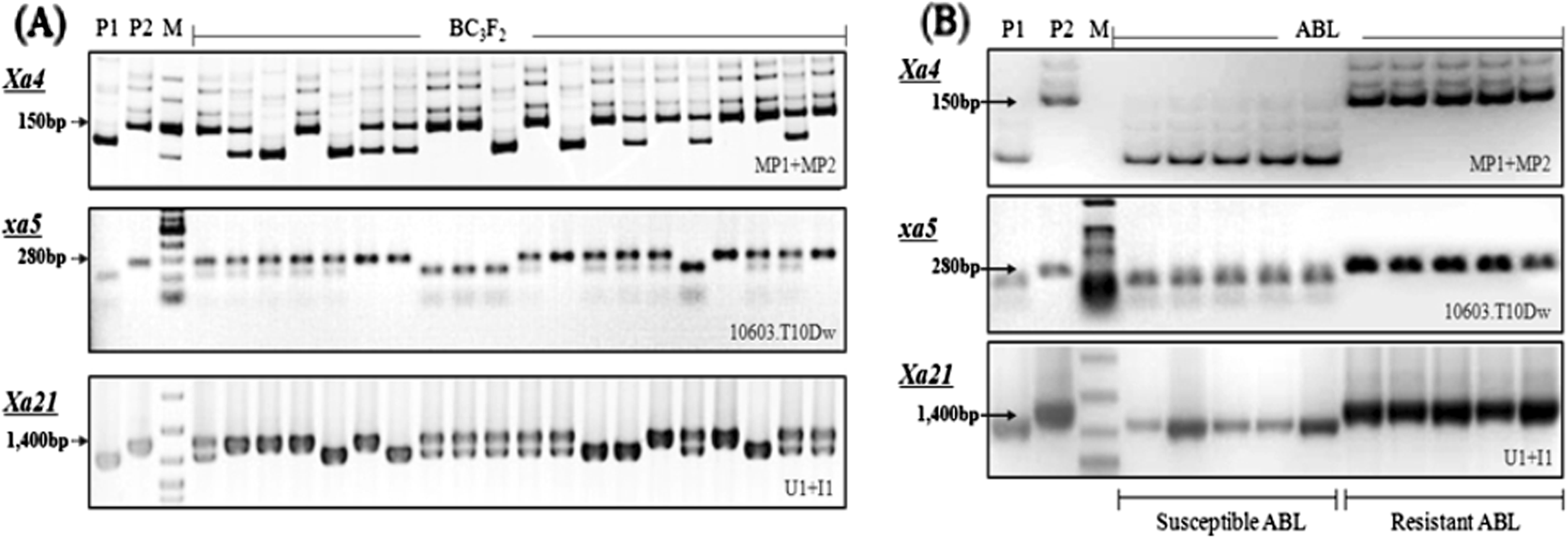

Supplement: Supplementary file 2 — Authors’ original file for figure 2 [file 12284_2012_42_MOESM2_ESM.tiff]

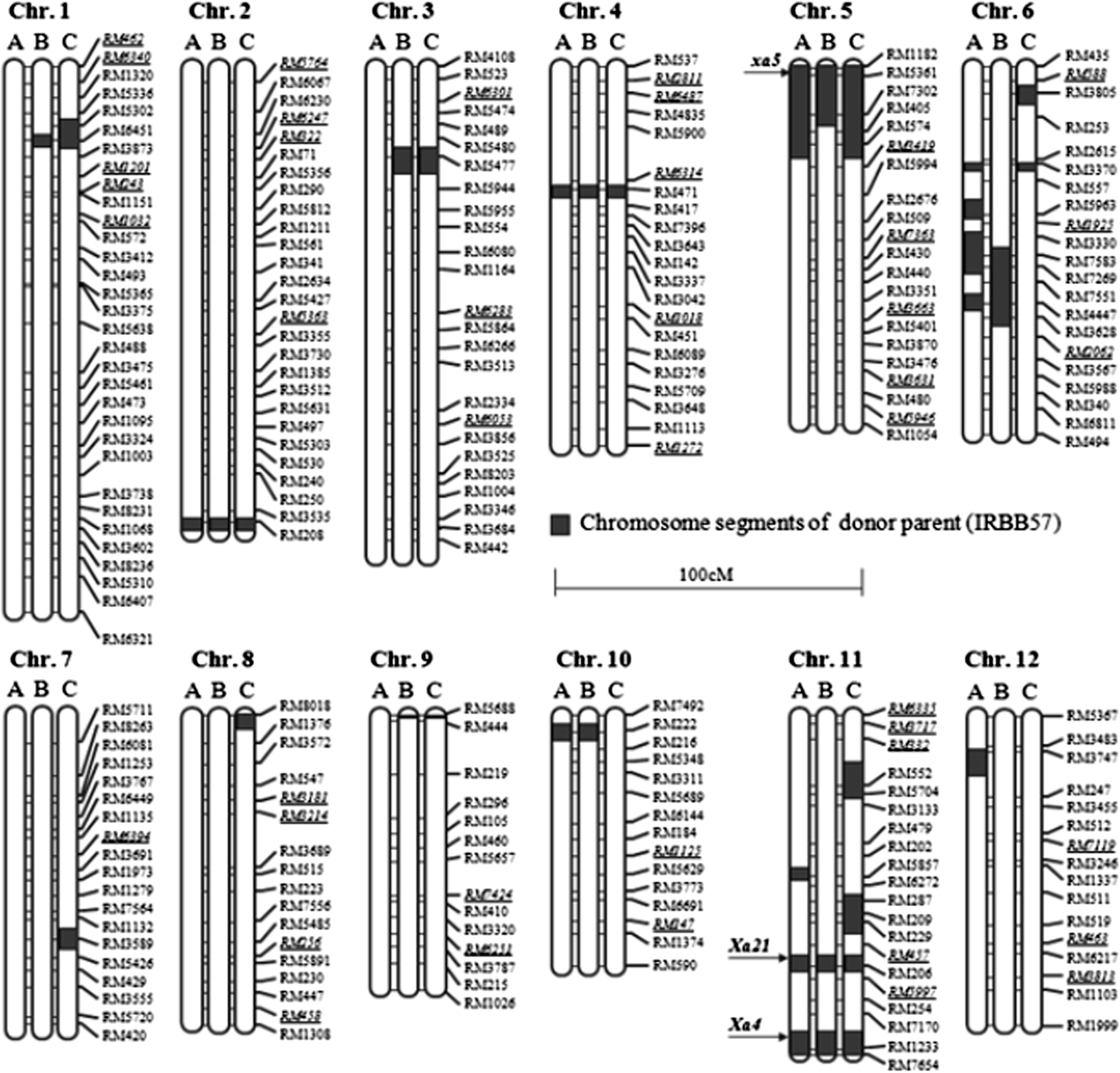

Supplement: Supplementary file 3 — Authors’ original file for figure 3 [file 12284_2012_42_MOESM3_ESM.tiff]
